# Are treatment benefits in neuropathic pain reflected in the self assessment of treatment questionnaire?

**DOI:** 10.1186/1477-7525-11-8

**Published:** 2013-01-18

**Authors:** Ingela Wiklund, Stefan Holmstrom, Malcolm Stoker, Kathleen W Wyrwich, Mary Devine

**Affiliations:** 1 United BioSource Corporation, 26-28 Hammersmith Grove, W6 7HA, London, UK; 2 Astellas Pharma Global Development, Elisabethhof 19, PO Box 108, 2350 AC, Leiderdorp, The Netherlands; 3 United BioSource Corporation, 7101 Wisconsin Ave, Suite 600, 20814, Bethesda, MD, USA

**Keywords:** Neuropathic pain, Content validity, Self assessment, Questionnaire

## Abstract

**Background/objective:**

The Self Assessment of Treatment (SAT) questionnaire was developed to reflect key patient reported outcomes of Neuropathic Pain (NP) treatments. This study aimed to understand how patients perceived the relevance and ease of understanding of the questions in the SAT and to recommend modifications based on patient and clinician interviews.

**Methods:**

Semi-structured interviews were conducted with clinicians and NP patients to provide information regarding treatment attributes and the impact of pain. Patients were debriefed on the SAT, a 5-item scale evaluating pain, activity level, quality of life (QoL) and satisfaction with treatment (recommend treatment and undergo treatment again). The SAT has a recall period reflecting back to the start of treatment. The qualitative analysis software ATLAS.ti 5.0 was used to analyze patient transcripts. Changes to the SAT were integrated into the questionnaire for a second round of debriefing interviews.

**Results:**

Three NP clinicians and 44 patients (20 painful diabetic neuropathy, 16 HIV-associated neuropathy and 8 post herpetic neuralgia) with a mean age of 60.3 (12.3) years and an even gender distribution were interviewed. Patient treatment experience included anticonvulsants (73%), antidepressants (34%), opioids (25%), and topical medications (41%). Pain descriptors and treatment attributes were similar across the three NP groups. Pain relief was judged the most important treatment attribute, followed by ability to undertake activities. Sleep improvement was another important attribute. Activity limitations and QOL were perceived as too broad and non-specific, and were split into 3 concepts each (activity limitations was split into self care, daily and physical activities and QOL was split into sleep, emotions, and social function). A 7-day recall period was introduced. The item stem and response options were made consistent, and a baseline and follow-up questionnaires were developed (except for the satisfaction items) to enable monitoring onset of treatment benefit and change over time.

**Conclusions:**

The content validity of the revised SAT was improved by the qualitative research, and NP treatment benefits are reflected in a more consistent fashion by the changes. Baseline and follow-up versions make it possible to perform assessments of change over time.

## Background

Neuropathic pain (NP) is one of the most prevalent pain etiologies [1]. It is a disorder of the peripheral nerves—the motor, sensory and autonomic nerves that connect the spinal cord to muscles, skin and internal organs [2,3], and is a complex type of pain which requires a demonstrable lesion as defined by the updated pain terminology and definitions provided by the International Association for the Study of Pain (IASP); ( http://www.iasp-pain.org/AM/Template.cfm?Section=Pain_Definitions) [4]. Recently, Treede and colleagues [5] redefined NP as “pain arising as a direct consequence of a lesion or disease affecting the somatosensory system.” Damage to the peripheral nervous system can occur as a result of several disorders including diabetes, infections such as herpes zoster (shingles) or HIV, trauma, and autoimmune disorders [1,6]. While NP can occur in any location, it often manifests in the extremities such as the hands, feet, or legs. The overall prevalence of NP varies from between 0.9 % to 8% in a general population [7,8]. Among HIV patients between 15% and 35% have NP, whilst the prevalence is 15% in patients with painful diabetic neuropathy or post herpetic neuralgia [9].

The NP symptom experience and the evolution of the condition are highly idiosyncratic with individual experiences varying widely between patients [1,6]. The course of NP can be episodic and recurrent, slowly progressing over many years, or it can quickly become severe and debilitating. Both direct and indirect costs of NP to society are substantial [10-14]. The consequences to the individual patient profoundly impair emotional and physical functioning with consequences such as movement limitation, an inability to perform major activities of daily living, needing help for personal care, frequent use of health care services, and work productivity losses [10,11,15,16]. The disease burden is also substantial as shown in a comparison of the world wide burden to patients of suffering from NP assessed in a cross national study [17]. The intensity of the pain as well as the duration of the pain contributes to the distress associated with NP.

Important outcome domains were identified in a survey of people with pain conducted by the Initiative on Methods, Measurement, and Pain Assessment in Clinical Trials (IMMPACT) [18]. These domains formed the basis of the IMMPACT group’s recommendations on a core set of outcome domains to be included in evaluation of treatment benefits [19]. Whilst pain relief measures are used to determine whether the patient has actually benefited from an intervention, and provide valuable information on how effectively pain is being managed, patient satisfaction measures capture the personal evaluation of the intervention provided [20].

Despite newer treatment modalities NP remains difficult to treat. Treatment of NP in the past mainly consisted of regular analgesics, suggesting patients are receiving suboptimal treatment options [21]. However, recently, the European Medicines Agency [22] and the US Food and Drug Administration [23] approved the use of a high-concentration capsaicin topical patch 8% (QUTENZA™). Capsaicin, in high concentrations like the QUTENZA™ patch, is a transient receptor potential vanilloid 1 (TRPV1) agonist that is useful in relieving pain. Whilst the QUTENZA™ EU label covers the treatment of peripheral NP in non-diabetic adults, either alone or in combination with other medicinal products for pain, the US label is licensed for the management of neuropathic pain associated with post herpetic neuralgia [22,23].

The five-item Self Assessment of Treatment (SAT) questionnaire was developed internally by Neurogesx for use in clinical trials to measure key areas of treatment benefit (i.e. pain, activities, quality of life and treatment satisfaction) recommended by IMMPACT [18]. Since psychometric documentation of the SAT was lacking, a post hoc analysis was conducted using trials data to validate the SAT [24]. The results indicated that the three pain and impact items were reliable and valid, whilst the satisfaction items performed less well [24]. The SAT has been used as a secondary end-point in two trials assessing treatment with Capsaicin in human immunodeficiency virus (HIV)-associated peripheral neuropathy (HIV-PN) with beneficial results for QUTENZA™ cited in the medical review conducted by the FDA [25], as well as by [26] for the treatment of post herpetic neuralgia [22].

The psychometric performance suggested that the SAT items could be further improved with additional patient input to ensure that items are clearly phrased, relevant and comprehensive, easy to understand, and that the response options and recall period are adequate to better assess pain related treatment benefits when used in clinical trials. Therefore, the purpose of this study was to better understand how well the SAT questionnaire reflects what patients perceived as relevant aspects of pain treatment and to document patient understanding of the individual SAT questions. Additionally, recommendations were provided on how to modify the instrument to be better suited to assess the benefits of pain treatment by changing the recall period, based on the information gained in the semi-structured patient interviews.

## Methods

### Clinician interviews

Clinicians were interviewed to derive information from the clinical perspective on the most relevant neuropathic pain symptoms as well as the benefits and drawbacks of neuropathic pain treatments. A total of 3 clinicians with expertise in pain therapy, anesthesia intensive care, neurology and endocrinology participated in one-on-one telephone interviews. On average, the experts reported seeing 20 patients per week with NP. This consultation/interview was conducted up-front to ensure that patient interview questions would be appropriately addressed in the semi-structured interview guide.

### Patient interviews

General inclusion criteria applied to all study participants were as follows: male or female; 18 years of age or older; presence of painful diabetic peripheral neuropathy (PDPN) or NP due to either post herpetic neuralgia (PHN) or painful HIV associated neuropathy (HIV-AN); presence of painful symptoms due to neuropathic pain with a numerical pain rating score (NPRS) of ≥4 at the time of screening; on a stable (not as needed, i.e., PRN) treatment for neuropathic pain for at least the past 3 months prior to screening; able to participate in a 90-minute, in-person interview; fluent in English (able to read and write); and willing and able to complete informed consent prior to study entry. Specific inclusion criteria for each pain group were as follows: PDPN, a diagnosis of painful, distal, symmetrical, sensorimotor polyneuropathy that is due to diabetes and made by the primary treating physician or Investigator at least one year prior to the screening visit; PHN, a diagnosis of PHN made by the primary treating physician or Investigator with at least 6 months of pain since shingles vesicle crusting; and HIV-AN, prior medical history of HIV-1 infection and prior diagnosis of HIV-DSP or antiretroviral toxic neuropathy documented by the consulting neurologist or investigator.

Exclusion criteria applied to all participants were as follows: parenteral opioid medication use; active substance abuse or history of chronic substance abuse within the past year, or prior chronic substance abuse (including alcoholism); neuropathic pain areas located only on the face, above the hairline of the scalp, and/or in proximity to mucous membranes; significant pain of an etiology other than one type of neuropathic pain (PHN, PDPN, or HIV-AN); significant ongoing pain from other cause(s) that may interfere with judging neuropathic pain, any implanted medical device (spinal cord stimulator, intrathecal pump, or peripheral nerve stimulator) for the treatment of neuropathic pain; recent history of a significant medical-surgical intervention; a clinically-relevant medical or psychiatric condition which, in the opinion of the investigator and/or coordinator, would interfere with completing the study including but not limited to sensory problems, cognitive impairment, acute mental illness, or inadequately treated depression or anxiety.

Because the SAT was developed for use in trials assessing the efficacy of the QUTENZA™ patch, efforts were taken to recruit a subset of patients who had experience with QUTENZA™ treatment. Similarly, efforts to recruit patients from the USA with a variety of socio-demographic backgrounds, as well as varied disease and treatment experiences, were implemented to ensure a diverse population was involved. Protocols were approved by an institutional review board, and patient consent was obtained prior to discussion of study related materials. Clinicians completed an enrollment and clinical form documenting each participant’s eligibility and treatment history.

During the semi-structured one-on-one qualitative interviews, participants were asked to describe their experience with NP, their treatment history, and how and why they value available treatment options. Interviews included questions and exercises designed to elicit participant discussion of salient pain treatment attributes and levels. Participants were asked to rate the importance of treatment attributes on a scale from 0 (Not Important) to 5 (Very Important). All interviews were led by a trained moderator. The interview guide included both structured and open-ended question.

During the interviews, patients were asked to complete the SAT and were then debriefed on each of the questions to uncover any issues with the SAT questions. The SAT was revised after the first round of interviews and additional debriefing interviews were conducted to ensure all changes were perceived as relevant improvements to the SAT.

### Measures

During the qualitative interview, in addition to the SAT, participants completed a NPRS, and a brief socio-demographic questionnaire with questions regarding age, gender, ethnicity, employment, education, and co-morbid conditions.

#### Self Assessment of Treatment (SAT) questionnaire

The SAT evaluation form included 3 questions with 5-point response options to assess the impact of treatment on three areas (pain relief; activity level; and quality of life), and 2 additional items regarding: 1) whether the patient would undergo the treatment again, and 2) a comparison of the study treatment to previous treatments for pain. For each question, the subject checked a box on a 5-point scale, where the middle option (0) indicated a neutral response and the lower (−2) and higher (+2) options indicated a negative or positive response, respectively. For example, SAT Item 1 asked the patient “How do you assess your pain relief after treatment in this study?” with the response options of “I feel my pain is much worse” (−2), “somewhat worse” (−1), “no better and no worse” (0), “somewhat better” (1), and “much better” (2); Question 2 “How do you assess your activity level after treatment?” with responses ranging from I feel much less active (−2), I feel somewhat less active (−1), I feel no more and not less active (0), I feel somewhat more active (1) to I feel much more active (−2); Question 3 “How has your quality of life changed after treatment?” I feel my quality of life is much worse (−2), I feel my quality of life is somewhat worse (−1), I feel my quality of life is no better and no worse (0), I feel my quality of life is somewhat better (1), I feel my quality of life is much better (2); Question 4 “Would you undergo this treatment again?” was “administered to subjects with only three response options: “No, absolutely not” (−2), “Unsure” (0), and “Yes, definitely” (2) and Question 5 “How do you compare the treatment in this study to previous medication or therapies for your pain?” The item performance and psychometric properties of the SAT have recently been examined [24].

#### The Numerical Pain Rating Scale (NPRS)

The NPRS is commonly used in pain medication trials [27] and is one of the many pain items in the modified Brief Pain Inventory (BPI) [28,29]. The NPRS is an 11-point graded scale ranging from 0–10 with 0 depicting “no pain at all” and 10 denoting “worst pain imaginable.”

### Data analysis

Descriptive statistics (mean, standard deviation and frequency) were used to characterize the interview sample in terms of sociodemographic data, health status, the numerical pain rating and clinical characteristics. All interviews were audio recorded with the participant’s permission and transcribed for analysis. The qualitative analysis was conducted by coding the transcripts using ATLAS.ti version 5.0 [30] software to organize the coded concepts using a coding dictionary developed by the coding staff as the coding process continued and concepts were identified. This process was adhered to systematically analyze, code, and compare quotes from participant interviews in order to evaluate the underlying structure of the qualitative data.

The information gained in the interviews was used to explore any potential relevant missing items and to provide recommendations on how to modify the SAT for use in future studies.

## Results

### Clinical expert input on neuropathic pain

According to the clinical experts, there is a high degree of variability in descriptions of NP experiences among patients with the same type of NP but the descriptions do not differ significantly across types of NP. Experts indicated that patients often report challenges with pain relief and there is a low efficacy rate with the existing NP treatments. Common oral medications prescribed for NP, such as anticonvulsants, opioids, and antidepressants provide continuing efficacy but the dose titration process can be time consuming and many patients experience side effects from oral medications. In contrast, topical medications offer a more agreeable side effect profile but the duration of efficacy is limited in comparison with the oral medications. Interestingly, the experts suggested that the QUTENZA™ patch may offer an extended duration of efficacy compared to other topical treatments for NP and it may be particularly beneficial for patients experiencing burning superficial pain with allodynia such as those with PHN or HIV-AN. Expert opinion also included some negative aspects of QUTENZA^TM^ such as painful application, or a lengthy application process requiring assistance from a medical professional.

### Patient demography and clinical characteristics

A total of 44 patients participated in the one-on-one qualitative interviews in California, Florida, Georgia, and Virginia over a 3-month period. Twenty patients suffered from PDPN, 16 had HIV-AN and eight had PHN. Demographic and clinical characteristics of the participants are provided in Table 1 and Table 2, respectively.

**Table 1:** Demographic characteristics and self report Co Morbid conditions

	**Total N=44**
**Mean Age, Years (SD) [Range]**	60.3 (12.3) [30.0-80.0]
**Gender (n, %)**	
Female	23 (52.3%)
Male	21 (47.8 %)
**Ethnicity (n, %)**	
Not Hispanic or Latino	38 (90.5%)
Hispanic or Latino	4 (9.5%)
**Race (n, %)**^**1**^	
White	31 (70.5%)
Black or African American	10 (22.7%)
Other^2^	3 (6.8%)
**Employment Status (n, %)**^**1**^	
Retired	18 (40.9%)
Disabled	14 (31.8%)
Part-time	5 (11.4%)
Full-time	3 (6.8%)
Unemployed	4 (9.1%)
Homemaker	1 (2.3 %)
Other^3^	1 (2.3%)
**Neuropathic Pain (n, %)**	
Mild	4 (9.3%)
Moderate	20 (46.5%)
Severe	11 (25.6%)
Very Severe	8 (18.6%)
**Health Conditions(n, %)**^**1**^	
Anxiety	7 (15.9%)
Arthritis	16 (36.4%)
Asthma	7 (15.9%)
Cancer	2 (4.6%)
COPD	11 (25.0%)
Depression	13 (29.6%)
Diabetes	18 (40.9%)
Hepatitis	2 (4.6%)
HTN	17 (38.6%)
Insomnia	4 (9.1%)
Back Pain	15 (34.1%)
Retinopathy	2 (4.6%)
Stroke	1 (2.3%)
Other^4^	11 (25.0%)
None	8 (18.2%)

^1^Participants were permitted to select more than one response.

^2^ Other racial backgrounds reported by participants included: European Mix (n=1); Mestizo (n=1).

^3^ Other employment reported by participants included: Not specified (n=1).

^4^ Other health conditions reported by participants included: AIDS (n=2); CHF, Adema, Neuropathy, Right Shoulder Torn (n=1); Emphysema (n=1); Heart attacks (n=1); Heart Disease (CAD), RLS (n=1); High cholesterol, indigestion (n=1); HIV (n=1); HIV, Gout (n=1); Migraines (n=1); Neuropathy (n=1).

**Table 2:** Clinical characteristics

	**Total N = 44**
**Mean of Patients’ Onset of Neuropathic Pain Diagnosis in Years (SD) [Range]**	7.5 (8.1) [1.0-40.0]
**Type of Neuropathic Pain (n, %)**	
Postherpetic Neuralgia (PHN)	8 (18.2%)
HIV-associated Neuropathy (HIV-AN)	16 (36.4%)
Painful Diabetic Peripheral Neuropathy (PDPN)	20 (45.5%)
**Mean of Patient’s Rating of Pain (SD) [Range]**	6.9 (1.7) [3.0-10.0]
**Frequency of Opioid Medication**^**1**^	
Hydrocodone	5 (11.4%)
Oxycodone	1(2.3%)
Other^2^	9 (20.5%)
**Frequency of Non-Opioid Medication**^**1**^	
Pregabalin	13 (29.6%)
Duloxetine	7 (15.9%)
Gabapentin	27 (61.4%)
Other^3^	24 (54.5%)
**Frequency of Topical Medication**^**1**^	
QUTENZA^TM^	11 (25.0%)
Lidocaine	3 (6.8%)
Other^4^	2 (4.6%)
**Mean Patient Height, feet (SD) [Range]**	5.5 (0.4) [4.5-6.2]
**Mean Patient Weight, pounds (SD) [Range]**	196.7 (45.9) [108.0-315.0]

^1^These categories are not mutually exclusive.

^2^Other Opioid medications reported by participants included: Darvocet (n=1); MS Contin (n=1); Tramadol (n=6); Vicodin (n=1).

^3^Other Non-Opioid medications reported included: Advil (n=2); Aleve (n=1); Amitriptyline (n=3); Aspirin (n=2); Baclofen (n=1); Carbamalbpine (n=1); Celebrex (n=1); Cymbalta (n=1); Diclofenac (n=1); Glipizide (n=1); Motrin (n=1); Naproxen (n=2); Nortriptyline (n=1); Topamax (n=3); Tylenol (n=1); Tylenol Extra Strength (n=2).

^4^Other Topical medications reported by participants included: Amitriptyline 5%, Gabapentin 5%, Ketamine 8% (n=1); Zotrix 0.75 Cream (n=1).

### Patient experiences with neuropathic pain

Participants reported experiencing many different types of pain and pain symptoms. The most prevalent pain descriptions included: burning, mentioned by 30% (n=13) of the participants, numb (n=12. 27%), sharp (n=10, 23%), pins and needles (n=9, 21%) and throbbing pain n=6, (14%). Less frequent pain descriptors characterized the pain as hot, stabbing, tingling, aching, dull, heavy, and shooting pain, whilst only one or two participants described the pain as cramping, electrical shock, freezing/cold, hypersensitive, biting, clamping, cutting, piercing, pinching, pressure, and tight. There was no major difference between the NP populations in the way they described their pain (Table 3).

**Table 3:** Pain descriptions

**Pain description**	**Illustrative quotation**	**PHN**	**PDPN**	**HIV-AN**	**All**
		**(n=8)**	**(n=20)**	**(n=16)**	**(N=44)**
Burning	02-102-1-0: It's always like burning, I don't know how else to say, but it feels like even clothes touching, it's burning. Apparently it's not, but that's what it feels like.	4	7	2	13
		(50%)	(35%)	(12%)	(30%)
	04-105-1-0: When it gets really bad and it will burn. Feel like its hot burning.				
Numb	05-102-3-0: It’s a tingling ah in your feet and it goes up to your um ankle and then it’s like numbness like you don’t feel nothing. You can be walking with flip-flaps and the next day you know you think you still have your shoes on, and you’re walking out in your bare feet.	0	8	4	12
		(0%)	(40%)	(25%)	(27%)
Sharp	01-102-2-0: It just comes on, I mean it’s like a sharp pain and then it just-I usually just try to walk it off or rub it off and then it goes away-and then it returns.	1	3	6	10
		(13%)	(15%)	(38%)	(23%)
	03-106-2-0: Uh, [clearing throat] it's-it's like when it's very severe it's like I'm-I'm walking on a bed of nails or thumb tacks and it, uh, just tingling and very, very, uh, very painful, sharp, sharp pain.				
Pins and Needles	01-101-2-0: Not so much pain as it was pins and needles and little stabbing and numbness.	1	6	2	9
		(13%)	(30%)	(13%)	(21%)
	03-103-2-0: A pinprick--you know, lots of pins, poking pins kind of a feeling kind of thing, so it's hard pain to describe, but that's as close as I can get.				
Throbbing	08-104-3-1: I think the fact that it's when it starts it is-it will kind of throb and throb and throb for a while.	0	2	4	6
		(0%)	(10%)	(25%)	(14%)

These pain symptoms had an impact on daily activities (Table 4) and aspects of participants’ personal lives. The most common impact of NP on daily activities reported by participants across all pain groups was that pain affected the ability to “do” or engage in daily activities, which was reported by 61% (n=27) of the participants with no difference across NP populations. Participants commonly reported that neuropathic pain interrupted daily activities and that they avoid doing certain activities as a result of their neuropathic pain.

**Table 4:** Impact of neuropathic pain

**Impact on daily activities**	**Illustrative quotation**	**PHN**	**PDPN**	**HIV-AN**	**All**
		**(n=8)**	**(n=20)**	**(n=16)**	**(N=44)**
Affects ability to do activities	**01-104-2-0**: I mean it-it-it’s bad. It hurts, uh, so bad that, you know, sometimes I can’t get up. I can’t walk.… You can’t do anything. And, you know, you do whatever and you just sit there and do nothing.	4	12	11	27
		(50%)	(60%)	(69%)	(61%)
	**03-102-2-0**: When I-when I go into, um-when I-when my pain starts, yeah, absolutely, I can't do anything. I can't think.				
	**05-104-30**: Um, because I can't really do the things that I really want to do.				
	**05-105-3-0**: Well, be-it bothers me because I can't get this stuff done I need to get done…And I couldn't cook for the children, I didn't go to the grocery store, so there's nothing really there for them to make when I'm not able to fix them something their selves. So that really bothers me because then I really got to get				
	up and, uh, it really hurts. You know what I'm saying, I got to get up.				
Interrupts Activities	**01-102-2-0**: If I’m trying to do something and it comes on, it interrupts what I’m doing and then, of course, I have to stop and wait ‘til it quits hurting and then continue with whatever I was doing. So, consequently, I don’t do near all the things that I used to do.	2	5	1	8
		(25%)	(25%)	(6%)	(18%)
	**02-101-1-0**: It really doesn't affect anything, I just have to stop certain things after a certain time.				
	**04-104-2-0**: Well, it stops me from doing a lot of, uh, walking and doing things that I'm doing, I have to stop.				
Avoid doing activities	**03-103-2-0**: I try not to walk much, I always try to find a way to sit down where it's best to put my feet up.	1	4	2	7
		(13%)	(20%)	(13%)	(16%)
	**06-101-2-1**: Well, if-if I'm going any place, you know, that I have to do any walking or-I tend not to-to go if I can help it, you know.				
	**08-101-3-1**: Well, socially and personally, it goes back to do-being able to do the little things that-uh, that causes me to be afraid.				

Participants were asked to provide examples of the activities affected by neuropathic pain (Table 4). Specific daily activities that were adversely affected by neuropathic pain included: shopping, work, cooking, driving or transportation, cleaning, and wearing clothing. Daily activities identified by 1 or 2 participants included: caring for someone else, going to appointments, home maintenance, running errands, and hygiene. The impact of neuropathic pain on daily activities was similar across pain types. During these discussions, sleep disturbance was commonly identified and had a negative impact among 20 participants (46%) across pain types.

The physical activities affected by neuropathic pain varied by NP population type. Forty five percent of participants with PDPN (n=9) and 56% of participants with HIV-AN (n=9) identified walking and 10% (n=2) and 19% (n=3) respectively identified standing, as physical activities commonly affected by NP, while none of the participants with PHN reported difficulty with these activities (Table 4). Gardening and yard work were reportedly affected by PHN (25% of participants, n=2) and PDPN (10% of participants, n=2) but not at all by HIV-AN participants. Exercise was only discussed as an affected physical activity by HIV-AN participants (n=5, 31%). Playing sports, (PHN n=2, 25%; PDPN n=5, 25%; HIV-AN n=3, 19%) and sitting (PHN n=2, 25%; PDPN n=1, 5.0%; HIV-AN n=3, 19%) were affected for participants in all pain groups, with 23% (n=10) of the participants identifying playing sports as a problem and 14% (n=6) of the participants identifying sitting as a problem. Other physical activities, mentioned by only 1 or 2 participants, included lifting and playing with kids. Participants in all pain groups reported an impact on social activities, but this impact was reported less frequently by participants with HIV-AN than by participants with PHN or PDPN.

In addition to the impact on activities, participants discussed the impacts of NP on their personal lives. About 27% (n=12) of participants across all pain groups noted a negative impact of NP on relationships quoting experiences such as “if you go somewhere with a friend or something and then you have to be-I’ll be like sitting there and then have the sharp pains and then they’re like oh, my gosh, what’s wrong with you? You know, it does interfere with your relationships.” Similarly, about 25% (n=11) of participants mentioned that pain had a negative effect on their emotional well-being. Only one or two participants reported experiencing changes in mental state, outlook on life, sex life, feeling short tempered and difficulty concentrating/focusing as a result of neuropathic pain.

Although descriptions of pain experiences and impacts were highly variable, the experiences reported by patients were not different across the NP populations. This similarity across pain types is consistent with the observations of high intra-pain type variability reported during clinician interviews.

### Treatment experiences

Patients were asked to discuss their experience with NP treatments. The most commonly reported type of treatment was anticonvulsant medication followed by antidepressants (Table 5). Participants also reported treatment with opioid and non-opioid analgesics such as NSAIDs and acetaminophen. Experiences with pain treatments other than oral medications included topical treatments (excluding QUTENZA™). Experience with topical treatments and the QUTENZA™ patch were more common among participants with PHN (n=5, 63% and n=2, 25%) whilst in HIV-AN 44% (n=7) used the QUTENZA™ patch in contrast to only 10% (n=2) and 5% (n=1) in PDPN patients. Physical therapy, acupuncture and injections were treatment options only reported by single individual patients.

**Table 5:** Current and prior neuropathic pain treatments

**# of participants**	**# of treatments**	**Anticonvulsant or anti-seizure medications**	**Antidepressant medications**	**Opioid medications**	**Non-Opioid Analgesic medications**	**Topical treatments**	**Qutenza patch**
Total	95	32	15	11	14	8	10
N=44		(73%)	(34%)	(25%)	(32%)	(18%)	(23%)
**PHN**	**23**	**5**	**3**	**4**	**1**	**5**	**2**
**N=8**		**(63%)**	**(38%)**	**(50%)**	**(13%)**	**(63%)**	**(25%)**
**PDPN**	**42**	**16**	**9**	**5**	**7**	**2**	**1**^**1**^
**N=20**		**(80%)**	**(45%)**	**(25%)**	**(35%)**	**(10%)**	**(5%)**
**HIV-AN**	**30**	**11**	**3**	**3**	**6**	**1**	**7**
**N=16**		**(69%)**	**(19%)**	**(19%)**	**(38%)**	**(6%)**	**(44%)**

^1^One PDPN participant was previous treated with Qutenza but did not recall the treatment. Therefore, this participant was not asked about the treatment experience.

During the discussion of current and prior treatments, participants were specifically asked to share their experiences with treatment use, availability, cost, and side effects. It is important to note that some participants have tried several different medications within a treatment type and reported different experiences for the different medications.

When asked to describe how easy or difficult it was to use each treatment, most participants (n=75, 82%) described treatments as easy to use. Anticonvulsant medications were generally reported as easy to use by between 73%–88% of participants across all pain groups, as were opioids (65–100%) and non-opioid analgesics (57%–100%). Most participants (78%–100%) with experience using topical treatments indicated that the treatments were easy to use. Half (n=5) of the participants that had tried the QUTENZA^TM^ patch said that it was easy to use.

During the discussion of treatment use, participants specified both positive and negative aspects of treatment that influenced their opinions on treatment usability. Pain relief, low pill burden, and ease of taking a pill were highlighted as positive benefits by a majority of the participants. Other participants mentioned duration of relief, improvement in sleep, ability to take the treatment with other medications, onset of relief, cost, and side effects. Twelve out of 16 participants (75%) with HIV-AN reported that pain relief was important in their assessment of treatment usability. There were few problems associated with oral medications although 4 participants (13%) commented on the high pill burden associated with anticonvulsants. More than half of the participants (n=25, 57.7%) reported changing neuropathic pain treatments at least once during the course of their treatment experience. The main reasons for switching were ineffective treatments (n=13, 50%) and side effects (n=11, 42.3%). Other reasons for discontinuing a previous therapy included: physician’s decision, insufficient pain relief, contraindication with another medication, high cost, high pill burden, too much trouble to use, short duration of effectiveness, medication taken off the market, messy application, and loss of effectiveness. The most common reason for starting a new medication was to provide additional pain relief (n=6, 23%). Participants also indicated that new mediations were added to on-going treatments to help with sleeping, break through pain, or severe pain.

In the rating of treatment attributes, pain relief (identified by 47% of the participants, n=45) was identified as the key aspect of treatment. By treatment, pain relief was named as the best aspect of treatment with the highest frequency for opioids (n=4, 64%), QUTENZA™ (n=4, 60%), and anticonvulsants (n=11, 50%). Other characteristics identified as “best aspects of treatment” included ease of use, no side effects, availability, cost, help with sleeping, duration of effectiveness, and quick on-set. “Reduction in pain” was rated as the most important attribute across all pain groups with an average score for all of = 4.7 (range 0–5). Among PHN patients the average was 5.0, PDPN4.6, and HIV-AN=4.8. Participants with PHN and HIV-AN rated ‘Duration of pain relief’ as tied with ‘reduction in pain’ as the two most important attributes in determining treatment success (average scores of 5.0 out of 5 and 4.8, respectively). The second highest rated attribute for participants with PDPN was “improvement in ability to do your usual activities” (average score = 4.5).

The most common treatment characteristic, irrespective of mode of administration, identified by participants as the “worst aspect of treatment” was side effects (28%). Pill burden was identified as the worst aspect for nine oral therapies discussed by participants.

Both clinicians and patients commented on topical as opposed oral medications with topical medication offering a more favorable side effect profile. These finding suggest that side effects play an important role when it comes to treatment satisfaction and its evaluation in clinical trials.

### Factors for treatment success

Characteristics of ideal treatments could be separated into 3 categories: results of treatment (n=40), route of administration (n=33), and treatment use (n=19). Ideal treatment attributes described by participants were generally similar across pain groups and focused on complete elimination of pain, permanent pain relief and no side effects. As to most favored routes of administration, taking a pill, use of a patch, and topical administration were reported in that order.

### Patient feedback on the SAT

During the debriefing interviews, several issues were reported related to the SAT. In question 1, the question stem and the response options presented challenges since they were not consistent, e.g., the question asks for an assessment of the pain level and the response option states the pain is worse/better after treatment. Question 2 asks respondents to assess the activity level after treatment. Participants found “activity level” to be too broad and unspecific and provided specific examples on activities affected by pain. Question 3 asks about changes in quality of life after treatment which generally was considered to be an unspecific concept. Similar to the question on activity levels, patients reported items they found were important to their quality of life. In question 4 where respondents are asked to state if they would like to undergo treatment again it became obvious that the response options did not fit the questions stem and were not consistent with responses to the questions. Question 5 asks respondents to compare the treatment to other pain treatments with responses describing level of preference for previous or current treatment. Hence the question stem and the response options did not match which caused problems for the respondents.

Additionally, the recall period for all items is retrospective requiring patients to reflect back to when treatment was started which is difficult to remember precisely and therefore fraught with recall bias. The use of positive and negative numbers to depict the response options, especially the negative numbers also caused confusion.

### Modifications of the SAT

The patient input suggested that items 2 (activity level) and 3 (quality of life) were too broad and nonspecific. Therefore, these questions were split into 3 sub-questions each using the detailed information derived in the qualitative research. The questions were revised to include examples of specific activities to describe examples of self care activities, daily activities and physical activities, all frequently mentioned in the interviews. With regards to question 3, the same procedure was followed replacing quality of life with specific aspects of your life using emotional well being, sleep and social function as illustrative examples, these again were derived from the interviews.

Questions 4 and 5 they were rephrased to “Based on your experience with the study treatment, would you like to receive this treatment again?” (no, definitely not–yes, definitely) and “Based on your experience with the study treatment, overall how does this treatment compare to other treatments you have received in the past?” (very much worse–very much better). The recommendation is to use questions 1–3 at baseline and predefined follow up visits, and to include questions 4 and 5 at the final visit only.

For questions 1**–**3, the recall period was changed to “Over the past 7 days.” The response options for questions 1**–**3 were changed to not at all/slightly better/moderately better/quite a bit better/very much better for all 3 questions to improve consistency.

Baseline and follow up versions of SAT questions 1–3 were also developed to enable assessment of response to treatment over time. Given the nature of questions 4 and 5 no baseline questions were developed for these items.

The changes made to the SAT to address the issues identified during patient interviews are summarized in Table 6.

**Table 6:** Summary of suggested revisions to the SAT

**Item**	**Original**	**Changes**	**Revised baseline item**	**Revised follow up and last visit items**
**Instructions**	Please mark one response for each question	Instructions to mark responses clarified.	**Instructions**: Please mark your response by marking one of the boxes for each question below.	**Instructions**: Please mark your response by marking one of the boxes for each question below.
**Item 1**	How do you assess your pain level after treatment in this study?	1. Item developed to assess pain level at baseline	1. Over the past 7 days, how would you rate your pain level?	1. Over the past 7 days, how much has the study treatment improved your pain level?
	- I feel my pain is much worse (−2)	2. Recall period changed to 7 days	- No pain at all	- Not at all
	- I feel my pain is somewhat worse (−1)	3. The response options were made consistent with the item stem and with other response options on the questionnaire	- Mild pain	- Slightly better
	- I feel my pain is no better and no worse (0)	4. Weighting numbers were removed	- Moderate pain	- Moderately better
	- I feel my pain is somewhat better (1)		- Severe pain	- Quite a bit better
	- I feel my pain is much better (2)		- Very severe pain	- Very much better
**Item 2**	How do you assess your activity level after treatment in this study?	1. Item developed to assess activity at baseline.	2. Over the past 7 days, how much has pain affected your ability to do the following activities:	2. Over the past 7 days, how has the study treatment improved your ability to do the following activities:
	- I feel much less active (−2)	2. Recall period changed to 7 days	A. Daily self care activities, such as showering and dressing?	A. Daily self care activities, such as showering and dressing?
	- I feel somewhat less active (−1)	3. The item was split into several questions that are more targeted to pain relief aspects noted during the qualitative interviews	B. Daily activities, such as cleaning, fixing things around the house, grocery shopping, preparing meals, going to appointments, caring for someone else and other day to day tasks?	B. Daily activities, such as cleaning, fixing things around the house, grocery shopping, preparing meals, going to appointments, caring for someone else and other day to day tasks?
	- I feel no more and not less active (0)	4. The response options were made consistent with the item stem and with other response options on the questionnaire	C. Physical activities, such as walking, exercising, gardening or yard work?	C. Physical activities, such as walking, exercising, gardening or yard work?
	- I feel somewhat more active (1)	5. Weighting numbers were removed	Response options for A, B, and C	Response options for A, B, and C
	- I feel much more active (−2)		- Not at all	- Not at all
			- Slightly	- Slightly better
			- Moderately	- Moderately better
			- Quite a bit	- Quite a bit better
			- Very much=	- Very much better
**Item 3**	How has your quality of life changed after treatment in this study?	1. Item developed to assess quality of life at baseline.	3. Over the past 7 days, how much has pain affected the following aspects of your life:	3. Over the past 7 days, how much has the study treatment improved the following aspects of your life:
	- I feel my quality of life is much worse (−2)	2. Recall period changed to 7 days	A. Emotional wellbeing such as mood, temperament or outlook on life?	A. Emotional wellbeing such as mood, temperament or outlook on life
	- I feel my quality of life is somewhat worse (−1)	3. The item was split into several questions that are more targeted to QOL aspects noted during the qualitative interviews	B. Ability to sleep?	B. Ability to sleep?
	- I feel my quality of life is no better and no worse (0)	4. The response options were made consistent with the item stem and with other response options on the questionnaire	C. Social functioning, such as participating in activities or relationships with friends and family?	C. Social functioning, such as participating in activities or relationships with friends and family?
	- I feel my quality of life is somewhat better (1)	5. Weighting numbers were removed	Response options for A, B, and C	Response options for A, B, and C
	- I feel my quality of life is much better (2)		- Not at all	- Not at all
			- Slightly	- Slightly better
			- Moderately	- Moderately better
			- Quite a bit	- Quite a bit better
			- Very much	- Very much better
**Item 4**	Would you undergo this treatment again?	1. This item now does not ask if the participant “would undergo” [treatment] as in the original question		4. Based on your experience with the study treatment, would you like to receive this treatment again?
	- No, definitely not (−2)	2. Weighting numbers were removed		- No, definitely not
	- No, probably not (−1)			- No, probably not
	- Unsure (0)			- Unsure
	- Yes, probably (1)			- Yes, probably
	- Yes, definitely (2)			- Yes, definitely
**Item 5**	How do you compare the treatment in this study to previous medication or therapies for your pain?	1. The item was rephrased to clarify the intended meaning of the question		5. Based on your experience with the study treatment, overall, how does this treatment compare to other treatments you have received for your pain?
	- Very much prefer previous (−2)	2. The response options were revised to fit the item stem.		- Very much worse
	- Somewhat prefer previous (−1)	3. Weighting numbers were removed		- Somewhat worse
	- No preference (0)			- No better no worse
	- Somewhat prefer this treatment (1)			- Somewhat better
	- Very much prefer this treatment (2)			- Very much better

## Discussion

There are a couple of pain satisfaction surveys. The American Pain Society (APS) Satisfaction Survey is one of these and has shown that satisfaction was influenced largely by effectiveness of medication, independent of pain intensity [31]. This suggests that a satisfaction survey related to the effectiveness of a pain medication is an important measurement tool for use in clinical trials of novel pain treatments which is also consistent with the recommendations of the IMMACT group [18].

Even though the APS Satisfaction Survey focused on pain management in general practice, it was not directly related to satisfaction with medication. The same applies to another pain survey, the Pain Treatment Satisfaction Scale (PTSS) [32] which measured satisfaction in patients receiving treatment for acute or chronic pain; it was not designed for use in clinical trials, as it evaluates satisfaction with medical care received as well as pain medication. Although a variety of instruments capturing patient satisfaction exists, none of these measures were identified as appropriate for assessing patient satisfaction with medication used to evaluate the effect of treatment of chronic pain in the context of a clinical trial, which led to the development of the SAT.

In all, forty-four patients across three NP populations were interviewed which can be regarded as a sufficiently large sample size. The consistency of the findings support content validity and concept saturation as defined by Leidy and Vernon [33]and Magasi et al. [34]. Specifically, they define saturation as the point at which no substantially new information/concepts continue to emerge beyond what has been previously mentioned when interviewing the last few patients. Additional support can be found in the FDA guidance document [35], “The number of patients is not as critical as interview quality and patient diversity included in the sample in relation to intended clinical trial population characteristics.” In general, the 3 NP populations were similar in the way they described pain and the impact of pain symptoms. Clinicians reported a high variability in terms of pain reporting across patients but not across different NP types. Although a small number of clinicians were interviewed for this study, they provided similar input on the variability of pain experiences. The pain descriptors used by patients also varied, but were consistent irrespective of NP type and very also similar to the descriptors included in the McGill short form pain questionnaire where pain is referred to as debilitating, burning, sharp, jabbing, deep, and aching, and can persist for months or years [36,37]. The similarity in pain description across all three NP types indicates that the content of the SAT is applicable for use across the different NP populations.

Similarities supporting the use of the SAT across all three NP populations prove that the concept of satisfaction with treatment assessment is fundamental in the evaluation of pain medication with both the clinical experts and the patients rating the reduction in pain as the most important treatment attribute followed by the ability to perform usual activities (by the PDPN population) and duration of pain relief (by the PHN and HIV-AN populations).

As pain reduction is unanimously identified as the most important aspect of treatment, it is not surprising that changes in pain have been shown to be closely linked to improvement in quality of life [13]. Once again, in the interviews, no difference was seen between the groups in terms of the impact of NP and the disruption of ability to perform daily activities and its impact on quality of life. With similarities in terms of pain definitions and similarities in the way that it affects individuals across all NP types as well as the fact that pain reduction is a critical component of treatment, it appears that the SAT would be appropriate for use in all NP types.

Pain is one area of research where the subjective experience and reporting by the patient is well-established and the concept of self assessment is particularly relevant. The importance of patient report/self assessment can be seen in work conducted by Taylor-Stokes and colleagues [38] where there was significant association between patient- and physician- rated severity but physician and patient ratings were discordant with physicians underestimating severity in 46.7% of patients who reported severe PDPN. Therefore, a patient-reported tool is critical in accessing the correct data. With patients identifying a reduction in pain as the most important treatment attribute, question 1 of the SAT is designed to assess this upfront. The patient interviews provided significant insight into the impact that pain has on patients’ lives and as a result questions 2 and 3 have been modified to reflect the multiple areas on which pain has an impact. Daily activities, physical activities, social activities and relationships were identified by participants with varying types and degrees of NP as facets of life that were affected by pain. These aspects are also captured by Ko and colleagues [39] who report that pain from PDPN interfered with general activity, mood, walking, normal work, relationships, sleep, and enjoyment of life. The addition of sleep and the emotional impact of pain identified by the participants are mirrored in the domain suggestions of the by the IMMPACT group [7,19,40]. The suggested modifications of the SAT enables patients to provide more details, as uncovered in the patient interviews, about the way NP affects their lives and the resulting impact of treatment in areas of life that matter to NP patients. Additionally, the consistency in pain and impact reports supports the content validity of the revised SAT, a critical component in any self report instrument [34,41,42].

With the introduction of baseline and follow up questionnaires and the inclusion of onset and duration of action of NP treatment, two important treatment attributes mentioned by patients in the interviews can be more efficiently captured. The modifications to the SAT provide an instrument that is more appropriate for use in clinical trials providing a baseline assessment and follow up assessments.

The previous recall bias is also amended and is more in line with FDA recommendations than the previous version as a result of the shorter recall period. The questions on the revised SAT are phrased in a consistent fashion to facilitate the respondents’ answering. Both the questions and the response options follow a similar pattern as well as the inclusion of examples derived from the interviews.

The content validity of the revised SAT has been supported by extensive qualitative research among diverse NP populations and clinicians and NP treatment benefits are now reflected in a more accurate and informed manner in the measure. Further alterations may be necessary once data on the measure performance has been collected but currently the most fundamental concept that of content validity has been assured. Without validity, a measure with good psychometric properties but not based on something real is not valid and has no use.

## Abbreviations

BPI: Brief Pain Inventory;EMA: European Medicines Agency;FDA: US Food and Drug Administration;HIV-AN: HIV associated neuropathy;IASP: International Association for the Study of Pain;IMMPACT: Initiative on Methods, Measurement, and Pain Assessment in Clinical Trials;NP: Neuropathic pain;NPRS: Numerical pain rating score;PDPN: Painful diabetic peripheral neuropathy;PHN: Post Herpetic Neuralgia;QoL: Quality of Life;SAT: Self Assessment of Treatment

## Competing interests

Stefan Holmstrom and Malcolm Stoker are full time employees of Astellas Pharma Global Development. The processing fees for this publication will be paid by Astellas Pharma Global Development. No other financial or non-financial interests to declare. Ingela Wiklund, Kathy Wyrwich and Mary Devine: Are employed by the United BioSource Corporation (UBC), which provides consulting and other research services to pharmaceutical, device, government and non-government organizations. In this salaried position, (staff) works with a variety of companies and organizations. He/She receives no payment or honoraria directly from these organizations for services rendered.

## Authors’ contributions

IW, KW, KD, SH and MS contributed to conception and design of the study, acquisition of data and interpretation of data; IW, KW and MD contributed to analysis and interpretation of data. All authors contributed to drafting the article or reviewing and revising it critically for important intellectual content; all authors approved the final version to be published. IW attests that the authors had access to all the study data, takes responsibility for the accuracy of the analysis, and had authority over manuscript preparation and the decision to submit the manuscript for publication.

## Funding source

Astellas Pharma Global Development provided funding for this study.
